# *Ophiorrhiza bibracteata* (Rubiaceae), a new species from Guangxi, China

**DOI:** 10.3897/phytokeys.275.189773

**Published:** 2026-05-29

**Authors:** You Nong, Gui-Yuan Wei, Hai-Yong Wei, Lie Long, Zhi-Rong Liu, Bin Feng, Ting Qin

**Affiliations:** 1 Guangxi Key Laboratory of Traditional Chinese Medicine Quality Standards, Guangxi Institute of Chinese Medicine & Pharmaceutical Science, No. 20–1 Dongge Road, Nanning, Guangxi, China Guangxi Key Laboratory of Traditional Chinese Medicine Quality Standards, Guangxi Institute of Chinese Medicine & Pharmaceutical Science Nanning China; 2 Guangxi Jinzhong Mountain Black-necked Pheasant National Nature Reserve, Longlin, Baise, Guangxi, China Guangxi Jinzhong Mountain Black-necked Pheasant National Nature Reserve Baise China; 3 Longlin Gezu Autonomous County State-owned Jinzhongshan Forest Farm, Longlin, Baise, Guangxi, China Longlin Gezu Autonomous County State-owned Jinzhongshan Forest Farm Baise China; 4 Guangxi Forest Inventory and Planning Institute, No. 14 Zhonghua Road, Nanning, Guangxi, China Guangxi Forest Inventory and Planning Institute Nanning China

**Keywords:** Morphology, new species, *
Ophiorrhiza
*, taxonomy

## Abstract

*Ophiorrhiza bibracteata* (Rubiaceae), a new species from northwest Guangxi, China, is described and illustrated. It can be distinguished from its allies *O. mitchelloides*, *O. nanlingensis* and *O. grandibracteolata* by the shape and size of the bracts (ovate, 8–11 mm long) and the shape and size of the calyx lobes (triangular, 0.8–1.0 mm long, glabrous, with glands between the lobes). According to the IUCN Red List Categories and Criteria, the new species is assessed as Data Deficient (DD) until more information becomes available.

## Introduction

*Ophiorrhiza* L. (Linnaeus 1753) is a genus within the Rubiaceae family that is rich in species and taxonomically complex. It encompasses approximately 200 to 300 species (Deb and Mondal 1997; Chen and Taylor 2011; Li 2020), currently has 384 accepted names (POWO 2026) and is primarily distributed across tropical and subtropical regions of Asia (Darwin 1976; Lo 1990; Deb and Mondal 1997; Chen and Taylor 2011; Deng and Huang 2012; Hareesh et al. 2015; Wong 2019; Hu et al. 2021; Schanzer and Nabatov 2022; Liu et al. 2023). This genus comprises species that are predominantly annual or perennial herbs, with sub-shrubs being a rare occurrence. *Ophiorrhiza* species can be readily distinguished by their obcordate (heart-shaped with the point at the top) and flattened fruits, which split open along a transverse slit at the apex into two valves (Darwin 1976; Lo 1990, 1999; Chen and Taylor 2011; Wu et al. 2019). Although the genus can be clearly characterised by its distinctive fruit shape, delineating individual species within it can occasionally prove to be quite challenging. This difficulty arises from the significant morphological variability amongst species (Nakamura et al. 2006, 2007; Duan and Lin 2007, 2009; Wu et al. 2017a) and the limited understanding of floral characteristics in the majority of species (Hooker 1880; Schanzer 2004; Wu et al. 2017b, 2017c). Although Razafimandimbison and Rydin (2019) transferred *Spiradiclis* Blume and *Keenania* Hook.f. to *Ophiorrhiza*, based on molecular phylogenetic evidence of paraphyly, we follow the traditional generic delimitation here. *Ophiorrhiza* is maintained as morphologically distinct from *Spiradiclis* by its obcordate, laterally compressed capsules dehiscing by two valves (vs. linear-oblong to subglobose capsules dehiscing by four valves in *Spiradiclis*).

China stands as a key diversification hub for the genus *Ophiorrhiza*. To date, around 72 species (including 50 endemics) of this genus have been documented within its borders. These species are predominantly found in southern and south-western China, with Guangxi Province and Yunnan Province being notable hotspots (Chen and Taylor 2011; Huang et al. 2017; Wu et al. 2017a, 2017b, 2017c, 2018; Tu et al. 2018; Duan et al. 2019; Wen et al. 2019; Hu et al. 2021; Liu et al. 2023; Shang et al. 2024; Xue et al. 2025).

Beyond its taxonomic diversity, the genus *Ophiorrhiza* holds significant medicinal value, which further underscores the importance of accurate species delimitation. Members of this genus are widely used in traditional medicine across tropical Asia to treat ailments ranging from snakebites and ulcers to inflammation and skin infections (Taher et al. 2020). However, the genus has garnered particular attention in modern pharmacology due to its ability to produce camptothecin, a potent monoterpene indole alkaloid with established anticancer activity (Roja 2008; Taher et al. 2020). Several *Ophiorrhiza* species, including *O. mungos* L. and *O. rugosa* Wall., have been identified as promising natural sources of this alkaloid (Roja 2006; Gopalakrishnan et al. 2013). Consequently, accurate identification of *Ophiorrhiza* species is not merely a taxonomic exercise, but a critical prerequisite for bioprospecting, conservation prioritisation, and the sustainable utilisation of medicinally valuable germplasm. In this context, the discovery and characterisation of new species contribute directly to both fundamental botanical knowledge and applied medicinal plant research.

During our field surveys in Guangxi Jinzhong Mountain Black-necked Pheasant National Nature Reserve, Longlin, Guangxi Zhuang Autonomous Region, in November of 2025, we found a particular *Ophiorrhiza* plant with flower buds in the forest. This species is unique in having two ovate persistent bracts. After consulting relevant literature (Tu et al. 2018; Duan et al. 2019; Razafimandimbison and Rydin 2019; Wen et al. 2019; Hu et al. 2021; Liu et al. 2023; Shang et al. 2024; Xue et al. 2025), checking relevant specimens and collecting the plant in February and March 2026, we confirmed that the unusual plant is new to science. It is described below.

## Materials and methods

The newly-discovered species was observed during fieldwork. The characters of the stem, leaf, petiole, inflorescence, peduncle, bract, calyx, corolla, ovary and style were studied. Voucher specimens were deposited at GXMI and IBK. For comparative morphology, we examined other *Ophiorrhiza* species using online images from the Chinese Virtual Herbarium (CVH, https://www.cvh.ac.cn/), Kew Herbarium Catalogue (http://apps.kew.org/herbcat/gotoHomePage.do) and JSTOR Global Plants (http://plants.jstor.org/). The description of the newly-discovered species was based on herbarium specimens. All measurements were made with a tape measure and callipers. The structure of the indumentum and its distribution were observed under a dissecting microscope at magnifications of more than 20×.

## Results

### Taxonomy

#### Ophiorrhiza bibracteata

Taxon classification

Plantae

GentianalesRubiaceae

Y.Nong & G.Y.Wei sp. nov

F4831F54-8200-5842-BDCB-D9435E61EFCC

urn:lsid:ipni.org:names:77380758-1

Figs 1, 2, 3

##### Type.

China • Guangxi: Longlin County, Jinzhongshan Town, Dazhai Village, 24°36'54"N, 104°57'50"E, in forest, elev. 1715 m, 5 February 2026, fl. *You Nong NY2026020501* (holotype: GXMI 051220!; isotypes: GXMI!, IBK!).

**Figure 1.**
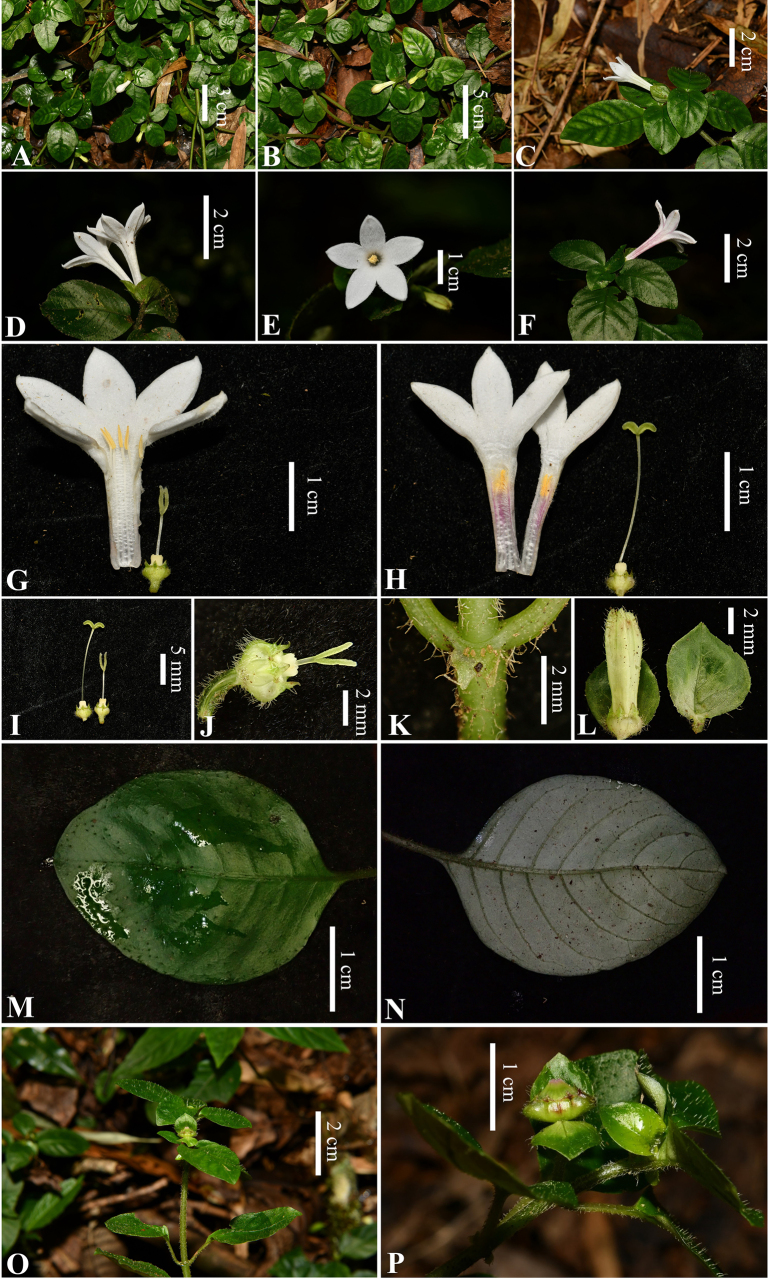
*Ophiorrhiza bibracteata* Y.Nong & G.Y.Wei. **A**, **B**. Habit; **C**, **D**. Flowering plant; **E**. Brevistylous flower (front view); **F**. Flower (lateral view); **G**. Dissected brevistylous flower; **H**. Dissected longistylous flower; **I**. Ovary, calyx, style and stigma of longistylous (left) and brevistylous (right) flower; **J**. Ovary, calyx, style and stigma of brevistylous flower; **K**. Stipule; **L**. Bracts and flower bud; **M**. Leaf, adaxial surface; **N**. Leaf, abaxial surface; **O**. Fruiting plant; **P**. Capsules and bracts (images captured with a Nikon D850 camera).

**Figure 2.**
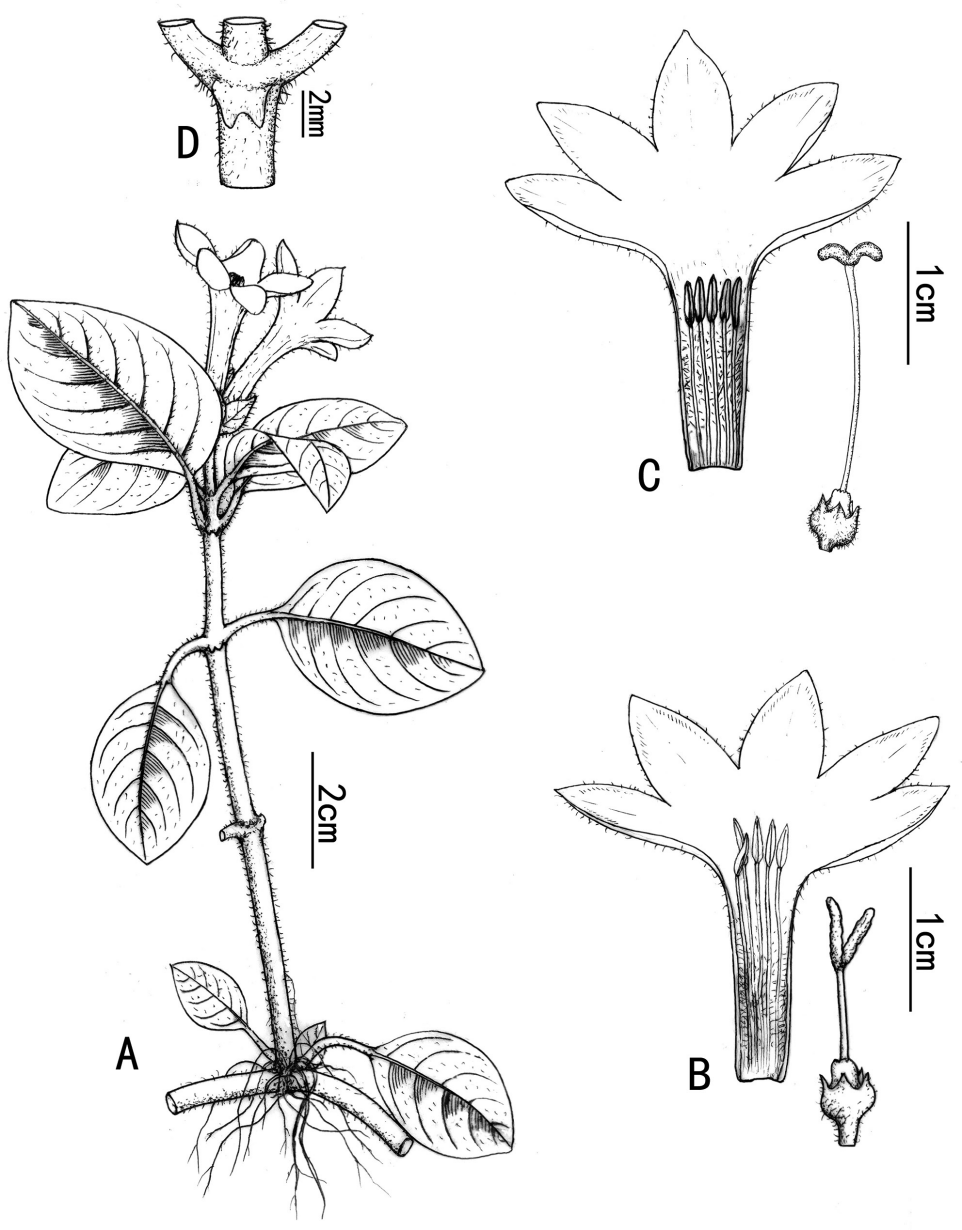
*Ophiorrhiza bibracteata* Y.Nong & G.Y.Wei. **A**. Flowering branch; **B**. Dissected brevistylous flower; **C**. Dissected longistylous flower; **D**. Stipule. Drawn by Xin-Cheng Qu from the voucher NY2026020501 (GXMI).

**Figure 3.**
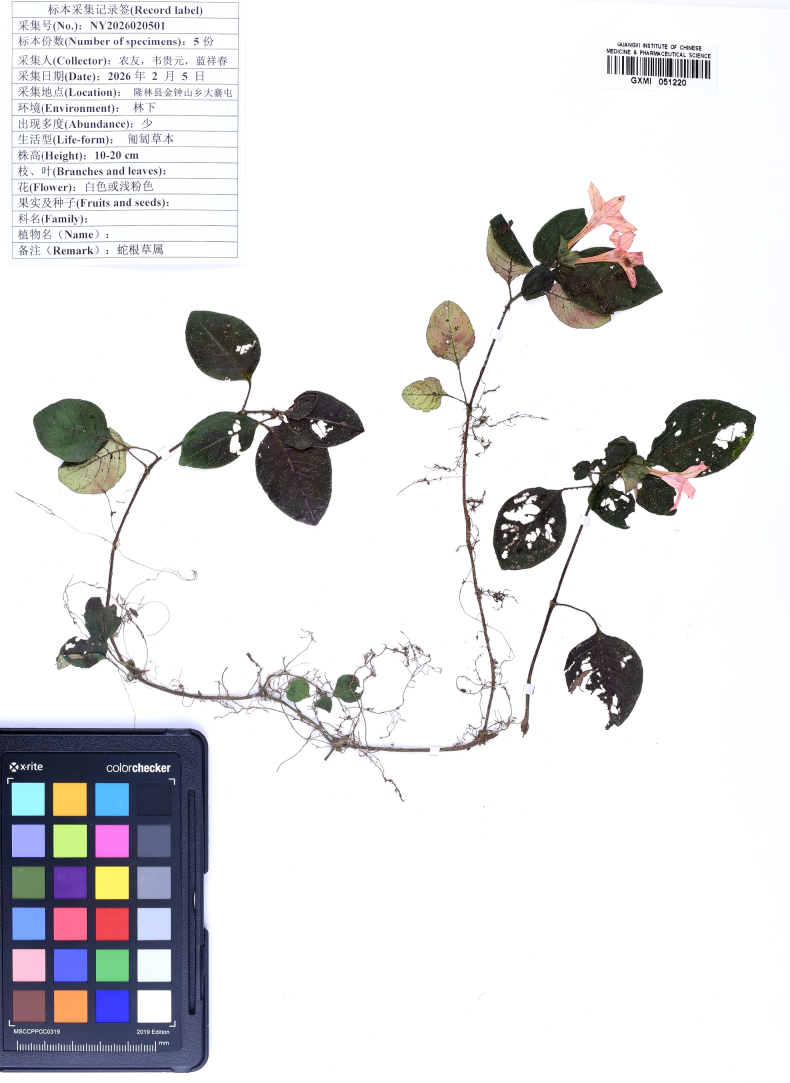
Type specimen of *Ophiorrhiza bibracteata* Y.Nong & G.Y.Wei.

##### Diagnosis.

*Ophiorrhiza bibracteata* is similar to *O. mitchelloides* (Masam.) H.S.Lo, but differs in the following characters: the number of secondary veins (5–7 pairs in *O. bibracteata* vs. 3–5 pairs), the shape and size of the bracts (ovate, 8–11 mm long vs. linear, 4–8 mm long), the shape and size of the calyx lobes (triangular, 0.8–1.0 mm long, glabrous, with glands between the lobes vs. linear, ca 1.4 mm long, glabrescent or ciliate, without glands) and the nature of the flowers (distylous vs. monostylous).

##### Description.

***Herbs***, creeping to ascending, to 20 cm tall, with most internodes prostrate, rooting at most nodes; stem densely pubescent with short brown unicellular trichomes, ca. 0.3 mm. ***Leaves*** in subequal to unequal pairs; blade drying papery to membranous, broadly elliptic or ovate, 1.5–4.5 × 1.5–3.0 cm, adaxially dark green and sparsely scabridulous, abaxially yellowish-green and sparsely pubescent along veins, obtuse at apex, broadly cuneate to rounded at base, margins entire, secondary veins 5–7 pairs. ***Petiole*** 0.6–2.2 cm long, pubescent. ***Stipules*** broadly ovate-triangular, folded backwards, glabrous, ca. 1 mm long, with stipitate glands at base inside, acuminate to 2-lobed. ***Inflorescence*** terminal, usually 1–2-flowered; peduncle 3–5 mm long, puberulent; bracts well developed, enclosing buds and at least partially flowers, persistent, ovate, 8–11 × 6–8 mm, pinnately veined, glabrous, except margins sparsely ciliolate. Flowers distylous, subsessile or pedicellate; pedicels 2–3 mm long. ***Hypanthium*** puberulent, 1.5–2.0 × 2.0–3.5 mm, 5-edged; calyx ± glabrous; lobes 5, triangular, 0.8–1.0 × 0.6–1.0 mm, with glands between the bases of the lobes. ***Corolla*** funnelform, pale pink or white, drying pink, glabrous outside, but sparsely puberulent on narrow longitudinal wings; tube 16–18 mm long, glabrous outside; lobes oblong-ovate, 8–12 × 6–8 mm, puberulent inside, dorsally narrowly winged, apex acute. ***Stamens*** 5; filaments short, glabrous; anthers linear, 2–3 mm long. ***Stigma*** bilobed; style glabrous; ovary 2-celled. ***Longistylous flower***: inside with a ring of white hairs at the middle of the corolla tube and puberulent from the middle up to the throat; stamens included, positioned near the middle of the corolla tube; filaments ca. 0.8 mm long; style 16–18 mm; stigma exserted from corolla tube, lobes ovate-elliptic, ca. 3 mm long. ***Brevistylous flowers***: sparsely pubescent at the middle of the corolla tube; stamens partly exserted, filaments ca. 3 mm long, inserted somewhat below the corolla throat; style 6–8 mm long, stigma reaching the middle of the corolla tube; stigma lobes lanceolate-elliptic, 3–4 mm long. ***Capsules*** mitriform, 6–8 × 5–6 mm, puberulent.

##### Geographical distribution.

**Figure 4.**
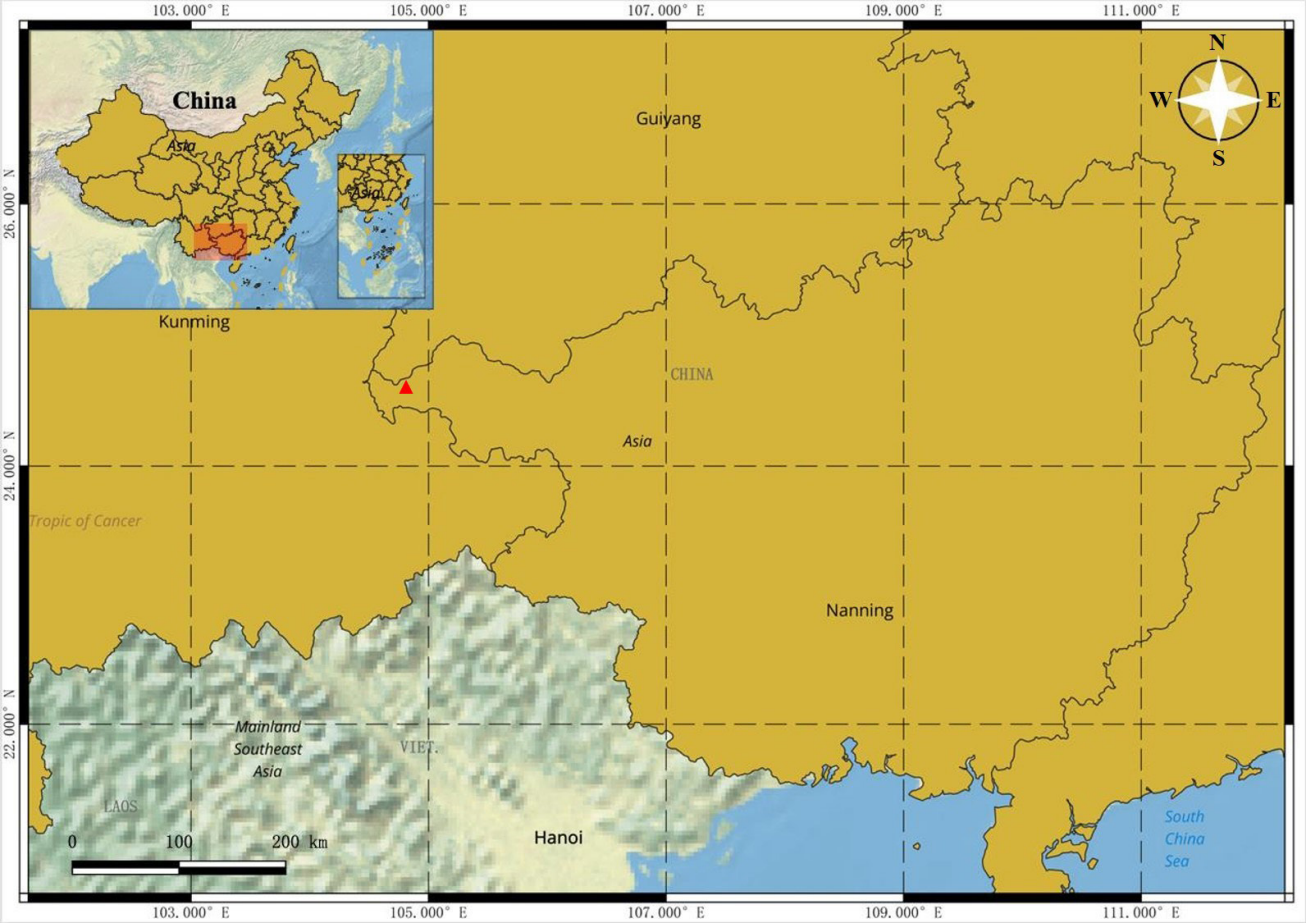
Type locality of *Ophiorrhiza bibracteata* Y.Nong & G.Y.Wei (red triangle) in Guangxi, China.

*Ophiorrhiza bibracteata* is found in Longlin County in Guangxi Zhuang Autonomous Region, northwest China (Fig. 4).

##### Ecology.

*Ophiorrhiza bibracteata* was discovered in moist places under subtropical evergreen broad-leaved forest at an elevation of 1715 m.

##### Phenology.

Flowering in February. Fruiting in March.

##### Additional material examined.

China • Guangxi: Longlin County, Jinzhongshan Town, Dazhai Village, 24°36'N, 104°57'E, in forest, elev. 1722 m, 15 November 2025, sterile, *You Nong NY2025111501* (GXMI!). China • Guangxi: Longlin County, Jinzhongshan Town, Dazhai Village, 24°36'N, 104°57'E, in forest, elev. 1722 m, 18 March 2025, fruiting, *You Nong NY2026031801* (GXMI!).

##### IUCN Red List category.

Data available for the new species are still insufficient to assess its conservation status. According to the IUCN Criteria (IUCN 2022), it is considered Data Deficient (DD) until more information becomes available. Further collection and monitoring are necessary to allow more conclusive estimates about the rarity and vulnerability of the species.

##### Etymology.

The specific epithet is derived from the Latin, bibracteata, meaning “two bracts” and referring to the two persistent ovate bracts of the new species.

##### Chinese name.

金钟山蛇根草 (jīn zhōng shān shé gēn căo).

##### Notes.

*Ophiorrhiza bibracteata* is similar to *O. nanlingensis* L.Wu & Q.R.Liu, but it differs in the shape and size of its bracts (ovate, 8–11 × 6–8 mm vs. subulate, 0.5–2.0 mm long), the nature of the flowers (distylous vs. monostylous); and the glabrous style (vs. sparsely pubescent at the middle).

*Ophiorrhiza bibracteata* resembles *O. grandibracteolata* F.C.How ex H.S.Lo in possessing broad ovate bracts, but it differs in the following characters: stems creeping to ascending, to 20 cm tall, rooting at most nodes (vs. ascending above, to 70 cm tall, most nodes not rooting); secondary veins 5–7 pairs (vs. 7–10 pairs); corolla pale pink or white, drying pink, tube 16–20 mm long; lobes oblong-ovate, 8–12 mm long (vs. corolla white or reddened, drying purplish-red; tube 22–25 mm; lobes subovate, ca. 5 mm long) and its looser inflorescence pattern (1- or 2-flowered vs. 5- to many flowered or rarely 1-flowered).

The main differences between the new species and the three other *Ophiorrhiza* species are summarised in Table 1.

**Table 1.** Morphological comparison amongst *O. bibracteata*, *O. grandibracteolata*, *O. mitchelloides* and *O. nanlingensis* (data from Chen and Taylor (2011); Hu et al. (2021)).

Morphological traits	*O. bibracteata*	*O. grandibracteolata*	*O. mitchelloides*	*O. nanlingensis*
Leaf blade	broadly elliptic or ovate, 1.5–4.2 × 1.5–3.2 cm; **secondary veins 5–7 pairs**	ovate, broadly ovate, or lanceolate-ovate, larger ones 2–15 × 1.2–6 cm; **secondary veins 7–10 pairs**	broadly ovate, ovate, or suborbicular, 0.8–2.5 × 0.6–2 cm; **secondary veins 3–5 pairs**	elliptic or ovate, 1.3–4.2 × 0.8–2.3 cm; secondary veins 5–7 pairs
Stipules	**broadly ovate-triangular**, ca. 1 mm long	**caducous**, not seen	**triangular to ligulate**, 1–1.5 mm long	broadly ovate-triangular, ca. 1 mm long
Inflorescence	**1- or 2-flowered**, villosulous	**5- to many flowered** or rarely 1-flowered, densely multicellular villous	**1- or 2(– 5)-flowered**, villosulous	**1–3-flowered**, puberulent
Bracts	**ovate, 8–11 × 6–8 mm**, glabrous, except margins sparsely ciliolate	**ovate to lanceolate, 10–15 mm**, pinnately veined, glabrescent, except multicellular ciliate along margin and on dorsal costa	**linear, 4–8 mm long**, villosulous	**subulate, 0.5–2.0 mm long**, subglabrous
Calyx lobes	triangular, 0.8–1.0 × 0.6–1.0 mm	triangular, 1–1.2 mm	**linear**, ca 1.4 mm long	triangular, 0.5–1.3 × 0.6–1.0 mm
Corolla	pale pink or white, glabrous outside, but sparsely puberulent on narrow longitudinal wings; **tube 16–20 mm long**; **lobes oblong-ovate, 8–12 mm long**	white or reddened, drying purplish-red, funnel-form, outside with 5 strigose lines from middle of tube to apices of lobes; **tube 22–25 mm long**; **lobes subovate, ca. 5 mm long**	white, outside with 5 strigose or hispidulous lines; **tube ca. 15 mm long**; **lobes broadly ovate, 5–6.5 mm long**	pale pink or white, glabrous outside; **tube 10–14 mm long**; **lobes oblong-ovate, 3–3.5 mm long**
Flower	distylous	distylous	monostylous	monostylous
Style	glabrous	glabrous	glabrous	**sparsely pubescent at the middle**
Capsules	mitriform, 6–8 × 5–6 mm, puberulent	rhomboid, 4–4.5 × ca. 11 mm, villous	obcordate, ca. 3.5 × 9–10 mm, villous	mitriform, 3.0–3.5 × 8–10 mm, puberulent

## Discussion

In recent years, molecular phylogenetic studies have provided valuable insights into the evolutionary relationships within *Ophiorrhiza*, helping to resolve some of the taxonomic ambiguities. However, comprehensive taxonomic revisions that integrate morphological, molecular and ecological data are still needed to fully clarify the species boundaries in this genus. In this context, the discovery and description of new *Ophiorrhiza* species are of great significance.

The morphological differences observed in the new species and its close relatives can provide valuable insights in the ecological adaptations and evolutionary history of the genus. The variation in bract size, shape and persistence may be related to differences in environmental factors, such as light intensity, humidity and herbivore pressure. For example, larger and more persistent bracts in some species might offer better protection to the developing flowers and fruits from herbivores or extreme weather conditions.

The new species, with its unique combination of morphological features, may represent an evolutionary branch that has adapted to a specific set of environmental conditions. Further molecular studies, such as DNA sequencing and phylogenetic analysis, would be beneficial to confirm or deny this evolutionary scenario and provide a more comprehensive understanding of the new species.

The following key includes nine species of *Ophiorrhiza* that share a procumbent to ascending habit, terminal inflorescences, relatively well-developed calyx lobes and a funnel-form corolla. These species are mainly distributed in southern China and exhibit a continuum of morphological variation in corolla tube length, bract development, leaf base shape and indumentum.

### Key to *Ophiorrhiza bibracteata* and its allies

**Table:** 

1	Inflorescence usually 1–2-flowered	***O. bibracteata***
–	Inflorescence usually with more than 3-flowers	**2**
2	Corolla tube relatively short, 7–12 mm long	**3**
–	Corolla tube longer, mostly 15–25 mm long	**5**
3	Bracts well developed, 3.5–6 mm long; leaf base cordate	***O. cordata* W.L.Sha**
–	Bracts reduced, 1–2 mm long; leaf base obtuse to truncate or cordulate	**4**
4	Corolla lobes 2.5–3 mm long	***O. dulongensis* H.S.Lo**
–	Corolla lobes 4–5 mm long	***O. huanjiangensis* D.Fang & Z.M.Xie**
5	Plants creeping to procumbent, rooting at most nodes	**6**
–	Plants erect to ascending, not rooting at nodes (or only at base)	**7**
6	Bracts linear or filiform, 1.5–10 mm long, not enclosing buds	***O. liangkwangensis* H.S.Lo**
–	Bracts ovate to elliptic, 5–12 mm long, often enclosing buds	***O. mitchelloides***
7	Bracts and bracteoles very large, 10–15 mm long	***O. grandibracteolata***
–	Bracts smaller, 3–7 mm long	**8**
8	Stems densely hispid or villous; leaves in markedly unequal pairs; corolla tube 18–22 mm long	***O. wenshanensis* H.S.Lo**
–	Stems glabrous to puberulent; leaves subequal; corolla tube 19–24 mm long	***O. nanlingensis***

## Supplementary Material

XML Treatment for Ophiorrhiza bibracteata

## Additional information

### Artificial Intelligence (AI) use

The authors accept full responsibility for the content of the manuscript, including the disclosure of any use of AI.

No AI tools were used in the preparation of this manuscript.

### Author ORCIDs

Y. Nong https://orcid.org/0000-0001-7004-0946

G.-Y. Wei https://orcid.org/0000-0003-0652-1213

Z.-R. Liu https://orcid.org/0009-0001-5171-5693

B. Feng https://orcid.org/0000-0003-0387-3635

### Data availability

All of the data that support the findings of this study are available in the main text.
